# A Comparison between Uni- and Bilateral tDCS Effects on Functional Connectivity of the Human Motor Cortex

**DOI:** 10.3389/fnhum.2013.00183

**Published:** 2013-05-07

**Authors:** Bernhard Sehm, Judy Kipping, Alexander Schäfer, Arno Villringer, Patrick Ragert

**Affiliations:** ^1^Department of Neurology, Max Planck Institute for Human Cognitive and Brain SciencesLeipzig, Germany; ^2^Clinic for Cognitive Neurology, University of LeipzigLeipzig, Germany

**Keywords:** tDCS, functional connectivity, primary motor cortex, unilateral tDCS, bilateral tDCS, interhemispheric, intracortical

## Abstract

Transcranial direct current stimulation (tDCS) over the primary motor cortex (M1) has been shown to induce changes in motor performance and learning. Recent studies indicate that tDCS is capable of modulating widespread neural network properties within the brain. However the temporal evolution of online- and after-effects of tDCS on functional connectivity (FC) within and across the stimulated motor cortices (M1) still remain elusive. In the present study, two different tDCS setups were investigated: (i) unilateral M1 tDCS (anode over right M1, cathode over the contralateral supraorbital region) and (ii) bilateral M1 tDCS (anode over right M1, cathode over left M1). In a randomized single-blinded cross-over design, 12 healthy subjects underwent functional magnetic resonance imaging at rest before, during and after 20 min of either bi-, unilateral, or sham M1 tDCS. Seed-based FC analysis was used to investigate tDCS-induced changes across and within M1. We found that bilateral M1 tDCS induced (a) a decrease in interhemispheric FC during stimulation and (b) an increase in intracortical FC within right M1 after termination of the intervention. While unilateral M1 tDCS also resulted in similar effects during stimulation, no such changes could be observed after termination of tDCS. Our results provide evidence that depending on the electrode montage, tDCS acts upon a modulation of either intracortical and/or interhemispheric processing of M1.

## Introduction

Over the past decades, non-invasive brain stimulation techniques have been used to investigate mechanisms of motor control and motor learning. Transcranial direct current stimulation (tDCS) utilizes low direct currents that are injected to the brain via surface electrodes. The effects of tDCS on the cortical tissue underlying the electrodes are highly polarity-dependent. For example, studies investigating excitability of the primary motor cortex (M1) showed that anodal tDCS leads to an increase of excitability within the stimulated area whereas cathodal tDCS (at least at an intensity of 1 mA) decreases cortical excitability. It has been suggested that such tDCS-induced brain alterations depend on changes in the neuronal membrane potential (Nitsche et al., 2003a, 2005).

In line with the capability of modulating excitability, anodal tDCS applied over M1 is capable of facilitating motor behavior and learning of the contralateral hand (Nitsche et al., 2003b; Reis et al., 2009). Usually, the “return” (cathodal) electrode is attached over the contralateral forehead and thought to be functionally ineffective. Recently, an alternative stimulation approach has been introduced that applied bilateral tDCS over M1 (Vines et al., 2008). Here, in addition to excitatory anodal stimulation of M1 contralateral to a hand exerting a motor task, the inhibitory cathode is placed over ipsilateral M1. Two studies directly compared the effects of both approaches in healthy subjects on performance or learning of unilateral sequential finger movements and yielded somewhat diverging results: while one study found an improvement in task performance only during bilateral M1 tDCS (Vines et al., 2008) another study did not find differences in implicit motor learning during bilateral as compared to unilateral M1 tDCS (Kang and Paik, 2011).

The concept of bilateral M1 tDCS has recently been adapted as an add-on therapy for neurorehabilitation in stroke patients with motor deficits. It has been suggested that bilateral M1 tDCS might not only facilitate neural activity in the damaged hemisphere but additionally helps to rebalance maladaptive interhemispheric interactions by inhibition of the contralesional M1. In this vein, a number of studies successfully showed the potential of bilateral M1 tDCS to enhance motor impairment in stroke patients (Lindenberg et al., 2010; Bolognini et al., 2011; Lefebvre et al., 2012). However, a direct comparison of the efficacy of bilateral and unilateral M1 tDCS has been missing so far in stroke patients.

The physiological mechanisms underlying the different tDCS setups are still largely unknown. Based on the concept of a functional and predominantly inhibitory balance between both M1 during unilateral hand movements (Perez and Cohen, 2008), it might be assumed that bilateral M1 tDCS interferes more prominent with interhemispheric mechanisms between both M1 as compared to unilateral M1 tDCS. However studies that focus on functional interactions within and between primary motor cortices induced by tDCS have been missing so far.

Combining tDCS and functional magnetic resonance imaging (fMRI) may be used to investigate online and after-effects of tDCS on a whole-brain level. Recent studies showed that tDCS is capable of inducing changes in functional connectivity (FC) both during and after stimulation in widespread brain networks (Polania et al., 2011a,b; Pena-Gomez et al., 2012; Sehm et al., 2012). Here we extend previous findings of our group that showed a dynamic modulation of whole-brain functional architecture both during and after tDCS (Sehm et al., 2012) by focusing on FC changes of M1. Specifically, we aimed at investigating changes in intracortical FC (ICFC) and interhemispheric FC (IHFC) of the primary motor cortex induced by unilateral and bilateral M1 tDCS as compared to sham. Furthermore we aimed at investigating the temporal dynamics of changes both during and after the application of tDCS. We hypothesized that both tDCS conditions would alter ICFC and IHFC of the primary motor cortex. Specifically, we expected an increase in ICFC in right M1 (the region of the anode in both conditions) and a decrease in IHFC between motor cortices. We further hypothesized that bilateral M1 tDCS would result in a stronger interhemispheric decrease as compared to unilateral M1 tDCS.

## Materials and Methods

### Subjects

Twelve healthy volunteers participated in the study (mean age 25.8 ± 3.2 SD; four female). All participants gave written informed consent to take part in the experiment in accordance with the declaration of Helsinki. The ethic committee of the University of Leipzig approved the study. All participants were healthy which was confirmed by a medical and neurological examination and were without any medication. Participants that had contraindications for tDCS or MRI measurements were excluded from the study. All participants were right-handed as assessed by the Edinburgh Handedness Inventory (Oldfield, 1971).

### Experimental procedure

**Figure 1:**
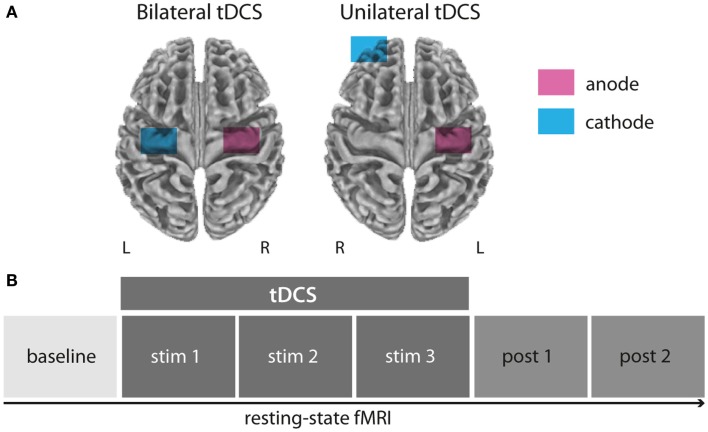
**Experimental setup and design**. All subjects underwent rs-fMRI during and after bilateral (anode over left, cathode over right M1), unilateral (anode over left, cathode over right supraorbital region), or sham M1 tDCS (not displayed) in three separated sessions **(A)**. Resting-state-fMRI measurements were conducted in six blocks before, during, and after tDCS application **(B)**.

Please also refer to Sehm et al. (2012) for a detailed description of all experimental procedures. In short, each subject participated in three sessions of concurrent tDCS and resting-state fMRI (rs-fMRI) in a cross-over design. Each session was separated by at least 1 week from each other to prevent any carryover effects. The experimental sessions only differed in the respective tDCS condition used: bilateral M1 tDCS (anodal electrode over right, cathodal electrode over left M1), unilateral M1 tDCS (anodal electrode over left M1, cathodal electrode over contralateral supraorbital region), or sham tDCS (here, the setup of either uni- or bilateral tDCS was randomly chosen). The order of sessions was counterbalanced across subjects. During each session, a total of six blocks rs-fMRI measurements were acquired: one baseline block without tDCS, three blocks during the application of tDCS (stim1–3), and two blocks after cessation of tDCS (post1–post2; Figure 1). Please see below a detailed description of scanning parameters and timing of the different experimental blocks.

### Transcranial direct current stimulation

For a detailed description of tDCS in the MRI environment please also refer to Sehm et al. (2012). In short, direct current was generated by a battery driven MRI compatible DC stimulator (Neuroconn GmbH, Ilmenau, Germany) that was positioned outside the scanner room. From the stimulator, the tDCS cables ran through a radio frequency filter into the MR cabin, where they were connected with the tDCS electrodes. The electrodes were equipped with 5 kΩ resistors in each wire to avoid sudden temperature increases and to reduce induction voltages due to high radio frequency pulses. Two filter boxes were placed between stimulator and electrodes inside and outside the scanner in order to reduce potential artifacts during image acquisition.

Transcranial direct current stimulation electrodes were wrapped in saline-soaked sponges and fixated to the participant’s head before the fMRI session outside of the scanner room using elastic bands. For unilateral M1 tDCS, the anode was centered over C4 according to the international 10–20 System while the cathode was attached to the forehead above the contralateral orbit. For bilateral M1 tDCS, the anode was again centered over C4 while the cathode was centered over C3 corresponding to the left M1 (Figure 1A). tDCS was delivered with a constant current of 1 mA during rs-fMRI. For all stimulation conditions (unilateral, bilateral, and sham M1 tDCS) the current was initially increased in a ramp-like fashion over 10 s, eliciting a tingling sensation on the scalp only during the first few seconds. During sham stimulation the current was turned off after 30 s.

### Resting-state functional MRI

We used a Siemens Magnetom Tim Trio 3 T scanner equipped with a standard eight-channel head coil for rs-fMRI measurements under eyes closed condition. Each session (total time of ∼55 min per session) consisted of six blocks of echo-planar-imaging (EPI): one block before tDCS (baseline; ∼7.6 min); three blocks during tDCS (stim1–3; ∼23 min); two blocks after tDCS (post1, post2; ∼15.3 min). During stim1–3, tDCS was applied for 20 min (unilateral and bilateral tDCS condition) or for the first 30 s only (sham condition). Each block was acquired with a total of 200 whole-brain volumes using the following parameters: acquisition matrix = 64 × 64, slice thickness = 3 mm (1 mm gap), voxel dimensions = 3 mm × 3 mm × 4 mm, 34 slices, TR = 2300 ms, TE = 30 ms, flip angle = 90°, bandwidth = 1825 Hz.

### Data analysis

We used a seed-based FC analysis to evaluate alterations in intracortical and interhemispheric M1 connectivity. A seed-based FC analysis is based on the anatomical hypothesis that tDCS applied at the scull over the left and right motor cortex influences the hemodynamic response in underlying and interconnected motor cortical brain regions. We chose two seed coordinates in the hand area of left and right precentral gyrus (±36, −21, −52) based on anatomical landmarks (Yousry et al., 1997) and a recent meta-analysis (Mayka et al., 2006). We masked this region with a sphere of 9 mm radius and extracted their averaged time series. The averaged time series was correlated voxel-wise with the entire brain. Afterwards, the correlation coefficients were Fisher’s *r*-to-*z*-transformed. We used 3dcalc implemented in AFNI (Cox, 1996) to contrast the individual *z*-maps of each experimental block (stim1–post2) to baseline in all three conditions (bilateral, unilateral M1 tDCS, and sham). The baseline-corrected difference maps were used as inputs for AFNI’s command 3dttest++ (with option -rankize). We conducted paired *t*-tests between stimulation conditions and sham for each of the five experimental blocks (stim1–3; post1–2) and masked left and right motor cortex using the Harvard Oxford atlas (thresholded at 25%) (Desikan et al., 2006). 3dClustSim was used for multiple comparison correction (Forman et al., 1995) and group-level *z*-maps were thresholded accordingly (*p* < 0.05, cluster corrected, voxel-wise *p* < 0.05, cluster size of 46 voxels as defined by Monte–Carlo simulation). For a direct comparison between unilateral and bilateral M1 tDCS effects, paired *t*-tests (corrected for multiple comparisons) were performed for time points, where significant differences could be observed for the respective tDCS condition relative to sham.

A control analysis was performed in the occipital pole using the same parameters as described above (seed coordinate 18, −104, 0). We chose this control region because it is distant from the cortex underneath the tDCS electrodes and had a similar signal-to-noise-ration of the rs-fMRI signal.

## Results

### tDCS-induced functional connectivity changes of the motor cortex (right M1 seed)

#### Bilateral M1 tDCS

**Figure 2:**
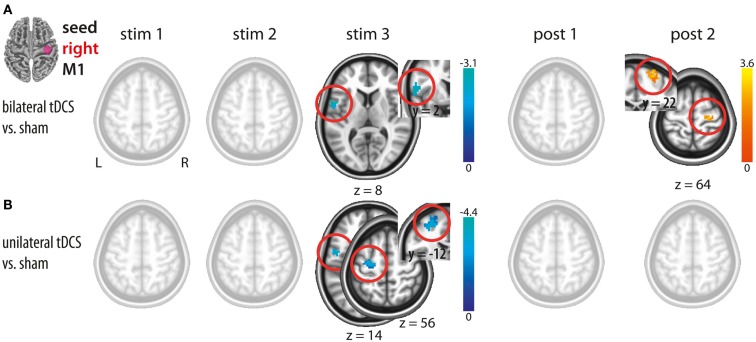
**Changes in functional connectivity during (stim1–3) and after (post1, post2) tDCS with the seed placed in the right M1**. **(A)** Bilateral tDCS vs. sham. **(B)** Unilateral tDCS vs. sham. Please note interhemispheric decreases during tDCS in both conditions and intracortical increase after bilateral M1 tDCS only. Significant clusters are presented on axial slices at a threshold of *z* > 1.96 (*P* < 0.05, corrected on cluster level). Color bars indicate *z* score range. L, left; R, right; M1, primary motor cortex.

Bilateral M1 tDCS as compared to sham resulted in a decrease in IHFC between right and left M1 during the last block of stimulation (stim3). This effect declined after termination of stimulation. However, during post 2 (∼8–15 min after termination of bilateral M1 tDCS), we observed an increase in ICFC within right M1. Thus, bilateral M1 tDCS induced (a) a decrease in IHFC during stimulation and (b) an increase in ICFC within right M1 after termination of the intervention (Figure 2A; Table 1).

**Table 1:** **tDCS-induced functional connectivity changes of M1**.

Experimental block	H	Coordinates (MNI)			*z* Max	Cl
		*x*	*y*	*z*	
**SEED RIGHT M1**						
**Bilateral M1 tDCS vs. sham**						
Stim3	L	−55	3	12	−2,62	53
Post2	R	26	−18	69	3,35	52
**Unilateral M1 tDCS vs. sham**						
Stim3, cluster 1	L	−28	−12	57	−3,55	90
Stim3, cluster 2	L	−52	3	15	−3,70	59
**SEED LEFT M1**						
**Bilateral M1 tDCS vs. sham**						
Post1	L	−58	−3	32	−2,90	69
**Unilateral M1 tDCS vs. sham**						
Stim3	L	−28	−15	60	−3,47	68

*M1, primary motor cortex; H, hemisphere; *z* max, maximum *z* value; Cl, cluster size*.

#### Unilateral M1 tDCS

In analogy to bilateral M1 tDCS, unilateral M1 tDCS as compared to sham resulted in a decrease in IHFC between right and left M1 during the last block of stimulation (stim3). In contrast to bilateral M1 tDCS, unilateral M1 tDCS did not affect FC within or between both M1s after termination of stimulation (Figure 2B; Table 1).

#### Comparison between uni- and bilateral M1 tDCS

A direct comparison of FC changes between bi- and unilateral M1 tDCS only resulted in differences in ICFC at post 2. This difference was driven by an increase in ICFC in the bilateral M1 tDCS condition. The comparison at stim3 did not result in any significant difference. Hence, our results provide novel evidence that the only detectable change in FC between both conditions is related to an increase in ICFC in right M1 induced by bilateral M1 tDCS.

### tDCS-induced functional connectivity changes of the motor cortex (left M1 seed)

#### Bilateral M1 tDCS

**Figure 3:**
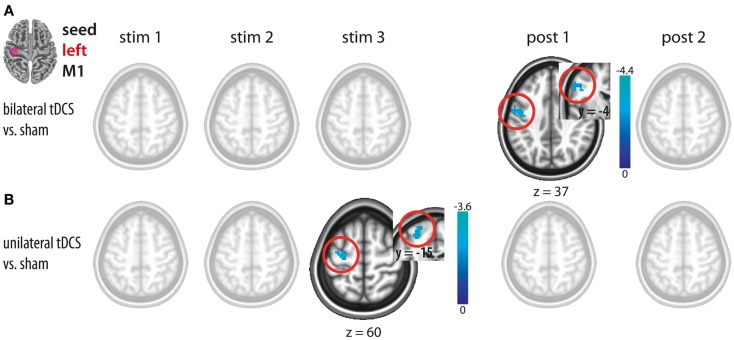
**Changes in functional connectivity during (stim1–3) and after (post1, post2) tDCS with the seed placed in the left M1**. **(A)** Bilateral tDCS vs. sham. **(B)** Unilateral tDCS vs. sham. Please note intracortical decreases within left M1 during unilateral and after bilateral M1 tDCS. Significant clusters are presented on axial slices at a threshold of *z* > 1.96 (*P* < 0.05, corrected on cluster level). Color bars indicate *z* score range. L, left; R, right; M1, primary motor cortex.

Bilateral M1 tDCS as compared to sham did not result in any FC changes within or between both M1. However, after termination of stimulation (post1), a decrease in ICFC in left M1 was observed (Figure 3A; Table 1).

#### Unilateral M1 tDCS

Unilateral M1 tDCS as compared to sham resulted in a decrease in ICFC within left M1 during stimulation (stim3), while no such changes could be observed after termination of unilateral M1 tDCS (Figure 3B; Table 1).

#### Comparison between uni- and bilateral M1 tDCS

A direct comparison between bi- and unilateral tDCS-induced FC changes for stim 3 and post1 revealed no statistical difference. These results indicate that although significant differences occurred when contrasting vs. sham, no such differences could be observed by a direct comparison between both tDCS conditions. Based on this, we postulate that there are no differences in ICFC within left M1 between both conditions.

In order to test for the specificity of the effect, we performed a control analysis in the occipital pole. No differences in FC were observed using the same parameters and analysis steps as in the motor cortex.

## Discussion

In the present study, we investigated online- and after-effects of tDCS on M1 ICFC and IHFC using rs-fMRI. Our findings demonstrate that tDCS is capable of modulating both ICFC and IHFC of M1, depending on the specific tDCS setup used. More specifically, we observed that both uni- and bilateral M1 tDCS resulted in a decrease in FC within left M1 and across left and right M1. As a distinguishing effect of both conditions, only bilateral M1 tDCS induced an increase in ICFC in right M1. This finding might reflect an additive effect driven by the cathodal electrode applied over left M1 during bilateral M1 tDCS. From a temporal perspective, interhemispheric changes occurred predominantly during bi- and unilateral M1 tDCS, while changes in ICFC within right M1 were exclusively observed after termination of bilateral M1 tDCS.

Despite efforts that have been undertaken in recent years to better characterize tDCS effects (Liebetanz et al., 2002; Nitsche et al., 2003a; Stagg et al., 2009; Fritsch et al., 2010; Marquez-Ruiz et al., 2012), the underlying biological processes are still largely unknown. The concurrent use of modern neuroimaging techniques and non-invasive brain stimulation might help to understand tDCS effects on a whole-brain scale (Stagg and Nitsche, 2011; Venkatakrishnan and Sandrini, 2012). Combining rs-fMRI and tDCS is a new approach to study tDCS-induced neuroplastic changes on a (whole brain) network level (Alon et al., 2011; Keeser et al., 2011; Polania et al., 2011b; Zheng et al., 2011; Pena-Gomez et al., 2012). A recent study from our group demonstrated dynamic online- and after-effects in whole-brain functional networks induced by tDCS (Sehm et al., 2012). In contrast, we here aimed at investigating more specifically the tDCS-induced effects on FC within and between the stimulated brain region (M1). Until now, only two other studies focused on tDCS-induced FC changes of the motor cortex using rs-fMRI: (i) Polania et al. (2012) showed, based on different graph-based FC parameters, that unilateral cathodal or anodal tDCS over the left hemisphere alters the functional architecture within left M1 in a pre-post design; (ii) another study investigated interhemispheric changes during unilateral anodal tDCS over the right M1 demonstrating a decrease in FC between right and left M1 (Alon et al., 2011), a finding that could be replicated in the present study (unilateral M1 tDCS condition). We extended this knowledge by investigating similarities and differences in FC changes induced by two different tDCS setups: bi- and unilateral M1 tDCS where we focused not only on tDCS-induced after- but also online-effects.

### Intracortical and interhemispheric changes in FC

**Figure 4:**
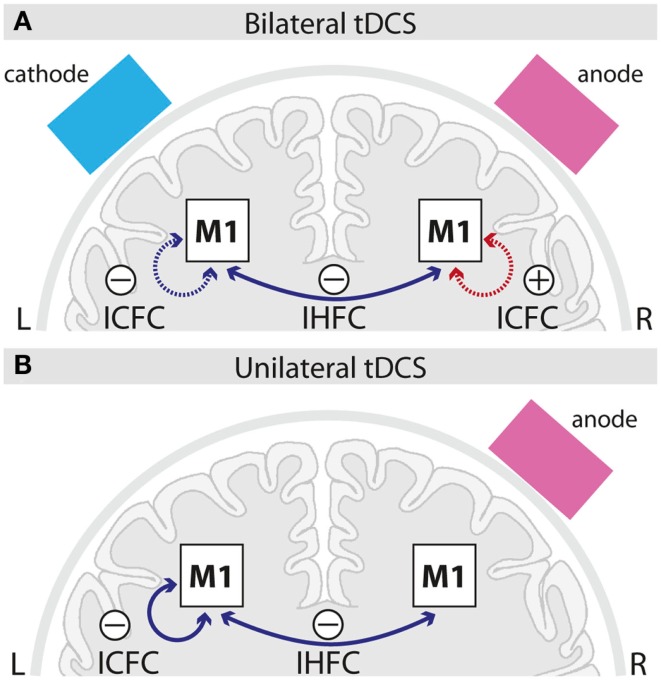
**Schematic summary of intracortical and interhemispheric functional connectivity changes induced by bilateral (A) and unilateral (B) M1 tDCS**. Solid lines indicate online-, dotted lines after-effects; blue lines indicate decreases, red lines increases in functional connectivity induced by the respective tDCS setup as compared to sham. ICFC, intracortical functional connectivity; IHFC, interhemispheric functional connectivity; M1, primary motor cortex; L, left; R, right.

Seed-based analysis allowed us to evaluate FC changes across hemispheres of the motor cortex (see Figures 2–4). The seeds of our analysis were chosen in the hand region of left and right M1, i.e., the cortical tissue underneath the tDCS electrodes. In this context, it should be noted that a recent current modeling paper suggested that the maximum field strength might not be necessarily maximal in the cortical tissue underneath the electrode (Miranda et al., 2013). However previous studies showed on physiological and behavioral level that application of tDCS using the same electrode montages like in the present study clearly impacts on the motor cortex (Nitsche and Paulus, 2000; Nitsche et al., 2003b; Vines et al., 2008; Reis et al., 2009). Here, we observed a decrease in IHFC in both tDCS conditions during the stimulation period, a finding that confirms but also extends previous findings using concurrent tDCS-rs-fMRI (Alon et al., 2011) (see Figures 2A,B). The decrease in IHFC was presumably based on a decrease in interhemispheric synchrony of low frequency fluctuations which in turn resulted in a functional decoupling between motor cortices. The effect was presumably driven by the anodal tDCS electrode attached over right M1, since this was the common component of both tDCS conditions. Our finding is in accordance with the general notion that non-invasive brain stimulation of M1 does not only alter the local cortical activity but also impact via transcallosal mechanisms on the opposite M1 (Reis et al., 2008). Contrary to our hypothesis, no differential effects on IHFC changes were observed between tDCS conditions. Thus, simultaneous facilitation of the right and inhibition of the homologous M1 has no additional effect on IHFC than facilitation of right M1 alone.

We further evaluated ICFC changes in M1, i.e., changes ipsilateral to the seed. A decrease in ICFC within left M1 was observed, even though during different time intervals, in both tDCS conditions (Figures 3A,B and 4). It seems unlikely, that this effect is specifically related to the cathode which was attached in different locations (left M1 vs. left supraorbital region) in both conditions, but rather could reflect an influence of the anode, that was positioned over the right hemisphere in both conditions, via transcallosal mechanisms. Only after bilateral M1 tDCS, however, an increase in ICFC in the right M1 was observed (Figures 2A and 4). This increase most likely reflects an additive effect of the cathodal electrode (positioned over left M1) on ICFC of right M1 via transcallosal pathways. It is tempting to speculate that this effect is presumably mediating the superior facilitation of motor performance after bilateral M1 tDCS (Vines et al., 2008).

It is yet an open question that should be addressed by future studies, whether the effects observed in our study may be specific to the hemisphere that was stimulated. In this vein, a previous study used excitability measurements with TMS to show that the effects of tDCS are dependent of the dominance of the targeted hemisphere (Schade et al., 2012).

While, to our knowledge, this is the first study investigating tDCS-induced interactions of FC within and across motor cortices, a number of studies used TMS-derived measures to investigate tDCS-induced excitability changes (Nitsche and Paulus, 2000; Lang et al., 2004; Nitsche et al., 2005; Williams et al., 2010; Mordillo-Mateos et al., 2012). The question remains whether or not connectivity and excitability measured with TMS (e.g., interhemispheric inhibition; IHI) and rs-fMRI (IHFC) reflect the same underlying neurophysiological mechanisms. This question yet remains unanswered and should be directly addressed by future studies using both, concurrent tDCS-rs-fMRI measurements and TMS-derived measures of excitability (Fox et al., 2012). Nevertheless, comparing our results with previous findings suggests that both measures might share a common neuronal substrate: a recent study provided evidence that bilateral M1 tDCS (in combination with a hand motor training) results in a decrease of IHI (Williams et al., 2010). Despite differences of the experimental design in both studies (electrode size; duration of stimulation; task-free vs. task design) it is tempting to speculate that the decrease in IHFC in the present study and the decrease in IHI as reported by Williams et al. (2010) during bilateral M1 tDCS might represent the same underlying physiological substrate.

### Temporal aspects

Our data provide evidence with respect to different temporal onset of neuroplastic changes in FC. While IHFC changes were observed during the last block of stimulation, an increase in ICFC (right M1) induced by bilateral M1 tDCS appeared only after termination of stimulation (Figures 2 and 4). In general, differences in timing might be caused by different latent intervals necessary to reveal the optimal strengthening of the synaptic efficacy resulting in an apparent change in FC.

The delayed effect on ICFC changes in the right M1 during bilateral M1 tDCS is of special interest in light of a study by Nitsche et al. (2004) that investigated the influence of pharmacological GABAergic modulation (using lorazepam) on the effects of motor excitability changes induced by (unilateral) anodal tDCS. After cessation of stimulation, they observed a delayed, but then enhanced and prolonged anodal tDCS-induced excitability elevation. Since intracortical mechanisms of facilitation or inhibition at that phase were unchanged, they argue that the prolongation of the excitability enhancement might be due to changes in remote brain regions – such as the contralateral M1 as in our study during bilateral M1 tDCS.

The pattern we observed with our experimental setup (uni- and bilateral M1 tDCS) might be specific to the stimulation duration used (20 min). Recently, it has been shown that 26 min of 1 mA anodal tDCS does not induce the expected facilitatory tDCS effect. Instead, prolonged stimulation times resulted in a tDCS-induced inhibition (Monte-Silva et al., 2012). Hence, it is tempting to speculate that the observed alterations in resting state might be different when stimulation durations other than the “standard” 20 min tDCS setup is used.

## Conclusion

Transcranial direct current stimulation, irrespective of the stimulation setup, interferes with IHFC across motor cortices. Bilateral, as compared to unilateral M1 tDCS exerts an increase in ICFC in right M1 after stimulation. Our results provide a framework of functional interactions within and across M1 in the healthy human brain induced by tDCS. From a clinical perspective, future studies are needed to investigate if and how tDCS alters motor cortical FC in the presence of disorders with maladaptive intracortical and interhemispheric mechanisms such as stroke.

## Conflict of Interest Statement

The authors declare that the research was conducted in the absence of any commercial or financial relationships that could be construed as a potential conflict of interest.
